# Combination of auraptene and arsenic trioxide induces apoptosis and cellular accumulation in the subG1 phase in adult T-cell leukemia cells

**DOI:** 10.22038/IJBMS.2021.58633.13025

**Published:** 2021-12

**Authors:** Mohaddeseh Kazemi, Hamideh Kouhpeikar, Zahra Delbari, Faeze Khodadadi, Sina Gerayli, Mehrdad Iranshahi, Arman Mosavat, Fatemeh Behnam Rassouli, Houshang Rafatpanah

**Affiliations:** 1 Immunology Research Center, Inflammation and inflammatory Diseases Division, Mashhad University of Medical Sciences, Mashhad, Iran; 2 Department of Hematology and Blood Bank, Tabas School of Nursing, Birjand University of Medical Sciences, Birjand, Iran; 3 Department of Pharmacognosy and Biotechnology, Biotechnology Research Center, Faculty of Pharmacy, Mashhad University of Medical Sciences, Mashhad, Iran; 4 Blood Borne Infections Research Center, Academic Center for Education, Culture, and Research (ACECR), Razavi Khorasan, Mashhad, Iran; 5 Novel Diagnostics and Therapeutics Research Group, Institute of Biotechnology, Ferdowsi University of Mashhad, Mashhad, Iran

**Keywords:** Adult T-cell leukemia/-lymphoma, Arsenic trioxide, Auraptene, Chemotherapy, MT-2 cells

## Abstract

**Objective(s):**

Despite advances in the treatment of adult T-cell leukemia/lymphoma (ATLL), the survival rate of this malignancy remains significantly low. Auraptene (AUR) is a natural coumarin with broad-spectrum anticancer activities. To introduce a more effective therapeutic strategy for ATLL, we investigated the combinatorial effects of AUR and arsenic trioxide (ATO) on MT-2 cells.

**Materials and Methods:**

The cells were treated with different concentrations of AUR for 24, 48, and 72 hr, and viability was measured by alamarBlue assay. Then, the combination of AUR (20 μg/ml) and ATO (3 μg/ml) was administrated and the cell cycle was analyzed by PI staining followed by flow cytometry analysis. In addition, the expression of *NF-κB (REL-A), CD44, c-MYC*, and *BMI-1* was evaluated via qPCR.

**Results:**

Assessment of cell viability revealed increased toxicity of AUR and ATO when used in combination. Our findings were confirmed by accumulation of cells in the sub G1 phase of the cell cycle and significant down-regulation of* NF-κB (REL-A), CD44, c-MYC, and BMI-1*.

**Conclusion:**

Obtained findings suggest that combinatorial use of AUR and ATO could be considered for designing novel chemotherapy regimens for ATLL.

## Introduction

Human T-cell leukemia virus type 1 (HTLV-1) is an etiologic agent of aggressive T-cell malignancy known as adult T-cell leukemia/lymphoma (ATLL) (1). Depending on the race and gender, almost 2–3% of patients infected with HTLV-1 develop ATLL, which is attributed to several factors, most notably the host immune response and HTLV-1-encoded viral genes (2-4).

HTLV-1-infected cells express viral Tax protein, which is involved in various cellular functions such as activation of nuclear factor kappa B (NF-κB), serine-threonine protein kinase (Akt) signaling pathways, and induction of cAMP, β-catenin, alongside with several anti-apoptotic proteins (5, 6). During the early stages of infection, Tax protein plays an indispensable role in the development of ATLL as it induces T-cell immortalization *in vitro *(7). It also inhibits the expression of *P53* and some cell cycle regulators and cyclins (8). Furthermore, it has been reported that Tax facilitates the migration and invasion of malignant cells via induction of angiogenesis and interaction between infected lymphocytes and endothelial cells (9). Tax-mediated *NF-κB* activation influences the expression of factors involved in cell migration, such as matrix metalloproteinase-9 (MMP-9), resulting in infiltration of infected T-lymphocytes (10). Indeed, *NF-κB* is a crucial signaling pathway in the proliferation and clonal expansion of HTLV-1-infected T cells as well as survival and proliferation of malignant ATLL clones (11). Other genes overexpressed in HTLV-1-infected cells are *CD44* and *c-MYC*, which are related to poor prognosis of ATLL patients. CD44 contributes to rearrangement of cytoskeleton and adhesion of T-cell lymphocytes (12). The CD44 marker expressed on skin-inﬁltrating tumor cells and CD44 soluble in the plasma reveal the severity of ATLL (13). The *c-MYC* proto-oncogene is a member of the MYC family that was initially known as the cellular homolog of a retroviral oncogene (*v-myc*) (14). c-MYC*,* involved in cell proliferation, apoptosis, and tumorigenesis, is associated with poor prognosis, progression, and invasion in human cancers (15, 16). BMI-1, a member of the polycomb group family of proteins, is another marker up-regulated in various cancers. *BMI-1* overexpression is associated with drug resistance, whereas its down-regulation might improve hematologic malignancies such as myelodysplastic syndrome, chronic myeloid leukemia, acute myeloid leukemia, and lymphoma (17).

Several chemotherapy regimens have been developed for ATLL treatment, as VCAP (consisting of the anti-cancer drugs vincristine, cyclophosphamide, doxorubicin, and prednisolone) and AMP (doxorubicin, ranimustine, and prednisone), although they cannot increase the survival rate of patients (18). Since current ATLL treatments, particularly in patients with acute and lymphomatous forms, do not lead to the control of malignancy and achievement of good response, there is a critical need for new therapeutic approaches (19).

Arsenic trioxide (ATO) has curative potential for treatment of acute promyelocytic leukemia, chronic myeloid leukemia, and a number of solid tumors (20). The anticancer effects of ATO may be related, at least in part, to induction of oxidative damage, G_1_ or G_2_/M phase arrest, and epigenetic regulation of miRNAs. Since toxic side effects of this drug have limited its medical applications, the potential of low doses of ATO in combinatorial approaches is under exploration (21). Auraptene (AUR), also known as 7-geranyloxycoumarin, is the most abundant prenyloxycoumarin that occurs in nature and can be synthesized in the laboratory as well. This monoterpene coumarin has gained a lot of attention because of its valuable anti-inflammatory, antioxidant, antibacterial, and anticancer properties (22, 23). Combinatorial effects of AUR with anticancer drugs, ionizing radiation, and hyperthermia have also been reported in various cancer cell types (24-27). In the case of leukemia, the anticancer effect of AUR has been indicated in Jurkat T cells that were mediated through a caspase cascade (26). However, to the best of our knowledge, there are no reports on the toxic effects of AUR on HTLV-1-infected human lymphocytes. Therefore, the purpose of this study was to determine the effects of AUR on MT-2 cells (an ATLL cell line), alone and in combination with ATO.

## Materials and Methods

***Cell lines and culture***

The HTLV-1-infected T-cells (MT-2 cell line) were obtained from the Pasteur Institute of Iran (Cell Bank of Iran, Tehran). MT-2 cells were cultured at 37 °C with 5% CO_2_ in RPMI-1640 medium (Biosera, France) supplemented with 10% fetal bovine serum (Gibco, USA), 0.5% penicillin/streptomycin and 0.5% L-glutamine (Gibco, USA). 

***AUR preparation***

AUR (7-geranyloxycoumarin) was synthesized via the reaction between 7-hydroxycoumarin (1 M) and trans-geranyl bromide (1.5 M) in the presence of 1,8-diazabicyclo[5.4.0]undec-7-ene (2 M) and potassium carbonate. AUR was purified by silica gel column chromatography, and after recrystallization, nuclear magnetic resonance spectra and melting points were obtained for product verification. AUR crystals (molecular weight 371 g/M) were then dissolved in dimethyl sulfoxide (DMSO, Germany) to prepare the first stock solution (20 mg/ml), and subsequently, it was diluted serially with DMSO to achieve other stock solutions with concentrations of 10, 5 and 2.5 mg/ml. Each stock solution was further diluted with culture medium to prepare 80, 40, 20, and 10 µg/ml, respectively. For control solution, 8 μl DMSO was added to 992 μl culture medium, and 50 μl of the resulting solution was added to 150 μl of the cell suspension in each control well. Thus, the solvent concentration in the control sample was similar to AUR concentrations, which was 0.2%. 

***ATO preparation***

To prepare various concentrations of ATO, 2 mg ATO powder (Sigma–Aldrich, USA) was added to 5 ml of distilled water, and sodium hydroxide was used to increase its solubility. Upon heating, the final volume was increased up to 10 ml, and 3, 6, 12, and 24 μg/ml ATO were prepared using a complete medium.

***IC***_50_ ***evaluation***

To determine the IC_50_ (half maximal inhibitory concentration) of AUR and ATO, MT-2 cells were seeded in 96-well culture plates (SPL, Korea) with a density of 50000 cells/well and treated with 10, 20, 40, and 80 µg/ml AUR, 3, 6, 12 and 24 μg/ml ATO and DMSO control solution for 24, 48, and 72 hr. After IC_50_ values were calculated, cells were treated with AUR and ATO alone and in combination at concentrations less than their IC_50_ for 72 hr. 

***Viability assay***

The alamarBlue assay was performed for quantitative analysis of cell viability. Upon each treatment, 20 μl of alamarBlue dye (Sigma, Germany) was added to each well, while three wells containing only 200 μl medium and dye were considered as blanks. Then, the optical density of wells was measured after 1, 2, 3, and 4 hr at 600 nm using a microplate spectrophotometer (Epoch, USA). Consequently, best results obtained at the optimal time were considered for calculation of cell viability using the following formula:

Percent of viability = 100 - (read number - negative control) / (blank - negative control) × 100

***Cell cycle analysis***

To study cell cycle changes, propidium iodide (PI) staining was applied. To do so, 72 hr after treatment with AUR, ATO, and AUR + ATO, alongside their relevant controls (DMSO and DMSO + ATO), cells were harvested and washed with phosphate-buffered saline (PBS). Next, 480 μl of PI staining solution, consisting of 350 μl PBS, 50 μl Triton X-100, 50 μl 0.1% sodium citrate, and 30 μl PI (100 μg/ml, Sigma, Germany), was added to cells and incubated at 37 °C for 30 min. Finally, samples were analyzed by flow cytometry (BD FACS Calibur, USA) using an FL2 filter.

***Quantitative polymerase chain reaction (qPCR)***

To evaluate the effects of AUR, alone and in combination with ATO, the qPCR method was applied. Briefly, the total RNA was extracted from cells treated with AUR, ATO, AUR + ATO, and their relevant controls using TriPure isolation reagent (Roche, Germany), and the purity of RNAs was assessed using a spectrophotometer (Thermo Scientific, USA). Then, cDNAs were synthesized using the RevertAid first-strand cDNA synthesis kit and oligo dT primer (Thermo Scientific, USA) according to the manufacturer’s instructions. To ensure the accuracy of amplified cDNAs, PCR was performed with all samples using GAPDH primers, and PCR products were analyzed by electrophoresis on a 1.2% agarose gel. qPCR was conducted with cDNAs to determine the expression level of *NF-κB* (*REL-A*) by TaqMan detection kit (Takara, Japan), while the expression of *CD44*, *c-MYC, *and *BMI-1* was evaluated using SYBR green PCR kit (Takara Biotechnology, Japan) according to the manufacturer’s protocol. The sequence of primers and probes used for qPCR are presented in Table 1. The PCR conditions for the TaqMan probe were as holding at 95 °C for 2 min, followed by 45 cycles of denaturation at 95 °C for 15 sec, annealing at 60 °C for 30 sec, and extension at 72 °C for 30 sec. PCR conditions for SYBR green method were as holding at 94 °C for 2 min, followed by 40 cycles of denaturation at 94 °C for 15 sec, annealing at 60 °C for 30 sec and extension at 72 °C for 30 sec. The relative quantity of target genes was normalized to that of β2-microglobulin as the reference gene and data were analyzed by standard curves relative method and reported as relative mRNA expression.

***Statistical analysis***

Results of viability assay were analyzed using GraphPad Prism Software Version 7 (GraphPad Software, Inc) and plotted as columnar graphs. Calculation of IC_50_ was carried out using Origin software. SPSS ver.16 software (SPSS, Chicago, IL) was used to analyze the results of qPCR. One-way analysis of variance (ANOVA) and Tukey test were performed to assess the statistical difference between groups, in which the *P*-value of each column was measured in comparison with its relevant control. Cell cycle analysis was evaluated using Win-MDI 2.8. Results are expressed as mean ±SEM and differences with *P*-values<0.05, <0.001, and <0.0001 were considered to be significant.

## Results

***Effects of AUR and ATO on the viability of MT-2 cells***

**Figure 1:**
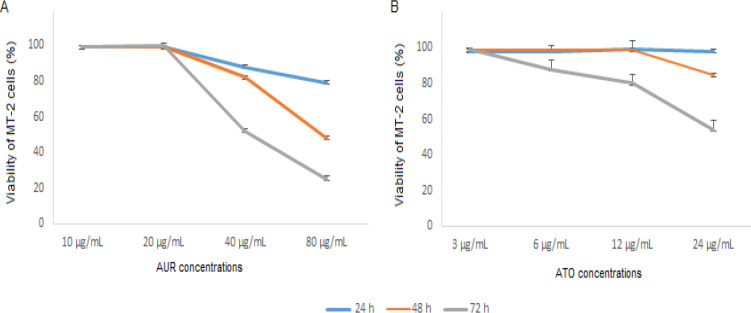
Viability of cells after treatment with AUR and ATO. MT-2 cells were treated with increasing concentrations of AUR (A) and ATO (B) for 24, 48, and 72 hr. Values are presented as mean ± SD

Assessment of viability indicated that AUR exhibited a cytotoxic effect on MT-2 cells in a dose-dependent manner (Figure 1A). The IC_50_ of AUR was calculated as 163.8, 74.2, and 49.5 µg/ml upon 24, 48, and 72 hr treatment, respectively. Moreover, the IC_50_ of ATO was determined as 1.3 mg/ml, 49.5 μg/ml, and 17.5 μg/ml after 24, 48, and 72 hr, respectively (Figure 1B). Accordingly, 20 μg/ml AUR and 3 μg/ml ATO were selected as less toxic concentrations of both agents for combinatorial treatments.

***Combinatorial effects of AUR and ATO***

**Figure 2:**
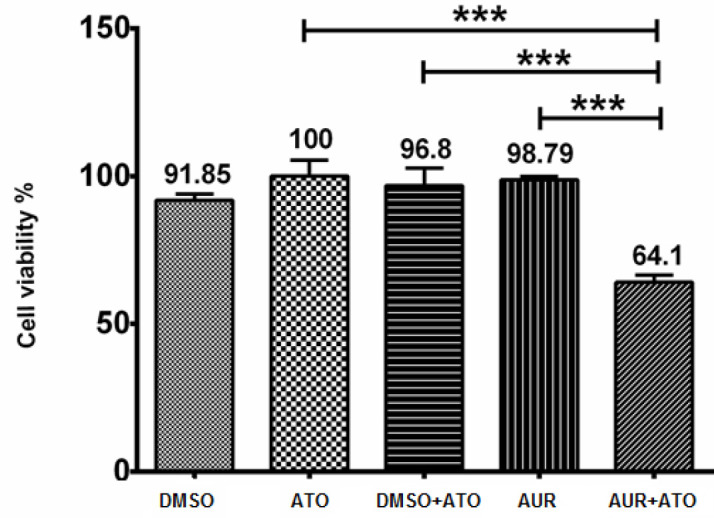
Assessment of cell viability upon treatment with AUR, ATO, and their combination. MT-2 cells were treated with AUR (20 μg/ml), ATO (3 μg/ml), AUR (20 μg/ml) + ATO (3 μg/ml), 0.2% DMSO and 0.2% DMSO + ATO (3 μg/ml) for 72 hr. Values are presented as mean ± SD. ***P<0.0001 compared with other treatments

ATO: Arsenic trioxide; AUR: Auraptene; DMSO, Dimethyl sulfoxide

**Figure 3:**
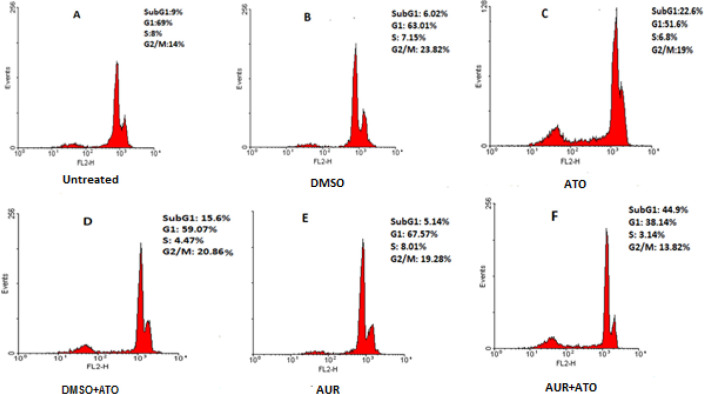
Cell cycle analysis after single and combinatorial use of AUR and ATO. PI staining and flow cytometry analysis were performed 72 hr after treatment of MT-2 cells with AUR (20 μg/ml), ATO (3 μg/ml), AUR (20 μg/ml) + ATO (3 μg/ml), 0.2% DMSO and 0.2% DMSO + ATO (3 μg/ml). Percentage of MT-2 treated cells with AUR + ATO in the sub G1 phase of the cell cycle was higher than those in other groups

ATO: Arsenic trioxide; AUR: Auraptene; DMSO, Dimethyl sulfoxide

To determine combinatorial effects of AUR and ATO, MT-2 cells were treated with AUR (20 μg/ml), ATO (3 μg/ml) and AUR (20 μg/ml) + ATO (3 μg/ml), alongside with their relevant controls including 0.2% DMSO, 0.2% DMSO + ATO (3 μg/ml) and untreated cells for 72 hr. As presented in Figure 2, the viability of cells in our combinatorial treatment declined significantly (*P*<0.0001) down to 64% in comparison with that in AUR (98.7%) and ATO (100%) treated groups. To confirm the observed effects of AUR + ATO treatment, cells were stained with PI and analyzed by flow cytometry (Figures 3A-F). As shown, upon AUR + ATO treatment, the percentages of cells in sub G_1_, G_1_, S, and G_2_/M phases of the cell cycle were 44.9%, 38.1%, 3.1%, and 13.8%, respectively. Nevertheless, in the control group (DMSO + ATO), the percentages of cells in the sub G_1_, G_1_, S, and G_2_/M stages were 15.6%, 59%, 4.4%, and 20.8%, respectively

***Alterations induced by AUR + ATO in gene expression***

**Figure 4:**
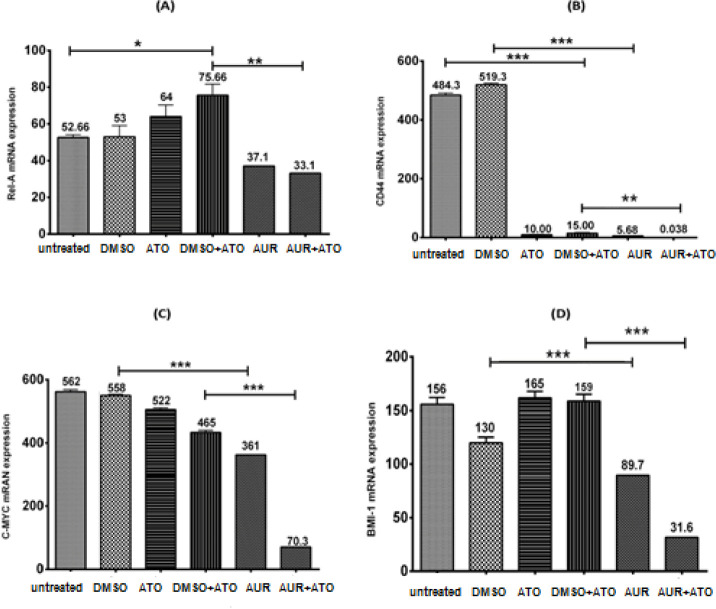
Gene expression analysis after single and combinatorial treatment with AUR and ATO. Expression of NF-κB (REL-A) (A), CD44 (B), c-MYC (C) and BMI-1 (D) was evaluated 72 hr after treatment of MT-2 cells with AUR (20 μg/ml), ATO (3 μg/ml), AUR (20 μg/ml) + ATO (3 μg/ml), 0.2% DMSO and 0.2% DMSO + ATO (3 μg/ml). Values are presented as mean ± SD. *P<0.05, **P<0.001, and ***P<0.0001 compared with relevant controls

ATO: Arsenic trioxide; AUR: Auraptene; NF-κB, Nuclear factor-kappa B; BMI-1, B cell-specific Moloney murine leukemia virus integration site 1; DMSO, Dimethyl sulfoxide

To investigate induced changes in the expression *NF-κB* (*REL-A*), *CD44*, *c-MYC*, and *BMI-1*, MT-2 cells were treated with AUR (20 μg/ml), ATO (3 μg/ml), AUR (20 μg/ml) + ATO (3 μg/ml) and their relevant controls for 72 hr. As depicted in Figure 4A, the expression of *NF-κB* (*REL-A*) decreased significantly upon AUR + ATO treatment (*P*<0.01) in comparison with that in DMSO + ATO control group. To note, *NF-κB* (*REL-A*) expression increased significantly (*P*<0.05) by DMSO + ATO in comparison with that in the untreated group. Besides, *CD44* expression exhibited a significant (*P*<0.001) decrease after treatment with AUR and ATO, alone and in combination (Figure 4B). As shown in Figure 4C, AUR and AUR + ATO significantly (*P*<0.001) reduced the expression of *c-MYC* when compared with their relevant controls. Last but not least, the expression of *BMI-1 *was significantly (*P*<0.001) down-regulated by AUR and AUR + ATO in comparison with that in their relevant controls, while ATO alone did not induce significant changes (Figure 4D). 

## Discussion

For most patients with ATLL, the use of current combined chemotherapy regimens is not sufficient to ensure long-term survival (17). ATO has shown inhibitory effects on hematological malignancies, however, it could not provide satisfactory outcomes due to its toxic side effects (20). Previous reports have indicated valuable anticancer effects of AUR, alone and in combination with various modalities (24, 28). Hence, to introduce a more effective therapeutic strategy for ATLL, we investigated the combinatorial effects of AUR and ATO on MT-2 cells. Results of the current study demonstrated that AUR could elevate the toxicity of ATO by 36% and cause cell accumulation in the sub G_1_ stage of the cell cycle. In addition, molecular analysis revealed a considerable reduction in the expression of *NF-κB* (*REL-A*), *CD44*, *c-MYC*, and *BMI-1* by our combinatorial approach.

The IC_50_ value of AUR in MT-2 cells was determined as 163.8, 74.2, and 49.5 µg/ml after 24, 48, and 72 hr, respectively. Epifano *et al*. evaluated the cytotoxicity of AUR on human colon cancer cell lines and reported that 10 μM AUR reduced cell survival to around 40% (29). In another study, treatment of human gastric cancer cells with AUR induced apoptosis via suppression of the mTOR pathway (30). It has also been reported that AUR induces apoptosis via activation of caspase 8 in Jurkat cells (28). The reported IC_50 _of AUR in colon, esophageal, breast, and gastric carcinoma cells, as well as T-cell leukemia cells, were 39, 76, 8, and 11 µg/ml, respectively (24, 25, 28, 30, 31).

Current findings revealed that combination of 20 µg/ml AUR + 3 µg/ml ATO significantly reduced MT-2 cell viability. Saboor-Maleki *et al*. reported that AUR elevated the sensitivity of esophageal cancer cells to cisplatin and 5-fluorouracil (31). Moreover, it has been demonstrated that AUR improves the cytotoxicity of cisplatin, doxorubicin, and vincristine on colon cancer cells (25). One of the reasons for drug resistance in cancer cells is the activity of membrane pumps that export chemical agents. It has been shown that AUR enhanced the accumulation of daunorubicin, a p-glycoprotein substrate, in multidrug-resistant human cervical carcinoma cells by competitively interacting with the drug-binding site of p-glycoprotein (32). Accordingly, it can be presumed that combination of AUR and ATO might augment the effect of chemotherapy in MT-2 cells via the same mechanism. 

Obtained results also verified that combinatorial use of AUR + ATO resulted in accumulation of cells in the sub G_1_ phase of the cell cycle, which represents dead cells with degraded DNA. Similarly, it has been reported that AUR induced cell cycle arrest in peripheral blood T lymphocytes, implying the anti-inflammatory and anti-proliferative properties of this natural coumarone (33). In addition, Moon *et al*. indicated that AUR induced accumulation of human gastric cancer cells in the sub G_1_ phase (30). Likewise, it has been shown that treatment of prostate cancer cells with AUR caused a noticeable augmentation in the sub G_1_ population (34). 

Investigating the molecular mechanisms involved in AUR + ATO effects revealed significant down-regulation of *NF-κB* (*REL-A*), *CD44*, *c-MYC*, and *BMI-1*. *NF-κβ*, as an important transcription factor, plays essential roles in angiogenesis, cell metastasis, proliferation, apoptosis, and chemo-resistance (35). HTLV-1 encodes the oncoprotein Tax-1, which constitutively activates *NF-κβ* via activation of IKK followed by degradation of IKB and transfer of REL-A to the nucleus (36). REL-A is an independent factor for predicting the survival and response time to the treatment in chronic lymphocytic leukemia (37). In patients with pancreatic cancer, the high expression of cytoplasmic and nuclear *REL-A* up-regulated *NF-κβ* expression, resulting in a lower survival rate among patients (38). In a mouse model of colorectal cancer, the use of AUR and its derivatives dramatically lowered the expression level of pro-inflammatory cytokines such as TNF-α, IL-1, and IL-6 and suppressed the activity of NF-κβ (39). Accordingly, down-regulation of *NF-κB* (*REL-A*) by AUR might explain, to some extent, improved cytotoxicity of ATO in ATLL cells.

*c-MYC* is a proto-oncogene that plays vital roles in cell apoptosis, adhesion, differentiation, proliferation, and metastasis, and its activity is highly regulated at transcriptional and post-transcriptional levels (40, 41). c-MYC is considered a target for designing effective anticancer approaches, and drugs that can suppress various cellular pathways regulated by c-MYC are attractive agents in anticancer therapy (42). Yang *et al*., reported *c-MYC* overexpression in gastric cancer cells upon cisplatin and 5-fluorouracil treatment, implying that increased expression of this gene is associated with drug resistance. They have shown that following the use of c-MYC inhibitor, 10058-F4, sensitivity of cancer cells to cytotoxic drugs was remarkably improved (17). Similarly, our findings indicated that AUR, alone and in combination with ATO, acts as a potent agent to reduce *c-MYC *expression in ATLL cells.

*CD44* and *BMI-1* are other genes that contribute to cell growth, differentiation, migration, metastasis, survival, and drug resistance (43). In a previous study performed on T-cell acute lymphoblastic leukemia (TALL) mice, it has been shown that *CD44* knock-down augmented chemosensitivity to doxorubicin and dexamethasone, suggesting that *CD44* is involved in drug resistance in TALL (44). On the other hand, it has been reported that AUR reduced *CD44* and *BMI-1* expression in esophageal and colon cancer cells (25, 31). Considering the importance of *CD44* and *BMI-1* in drug resistance, the findings of the present study suggest that AUR could be considered an appropriate option to cope with drug sensitivity of ATLL cells. 

The current study has some limitations: to validate cell experiments and elucidate the exact molecular mechanism of AUR action, these measurements should be performed and compared with other ATLL cell lines and be confirmed at the protein level.

**Table 1:** The primers and probes sequences of BMI-1, C-MYC, CD44, REL-A, GAPDH and β2-microglobulin genes

**Products size (bp)**	**Sequence (5'→3')**	**Genes**
**192**	Forward: CTGCAGCTCGCTTCAAGATG Reverse: CACACACATCAGGTGGGGAT	***BMI-1***
**159**	Forward: ACTCTGAGGAGGAACAAGAA Reverse: TGGAGACGTGGCACCTCTT	***C-MYC***
**176**	Forward: CGGACACCATGGACAAGTTT Reverse: GAAAGCCTTGCAGAGGTCAG	***CD44***
**145**	Forward: ACCCCTTCCAAGTTCCTATAGAAGAG Reverse: CGATTGTCAAAGATGGGATGAGAAAG Probe: FAM-ACTACGACCTGAATGCTGTGCGGCTCT-BHQ-1	***REL-A***
**101**	Forward: GGAAGGTGAAGGTCGGAGTCA Reverse: GTCATTGATGGCAACAATATCCACT	***GAPDH***
**127**	Forward: CTTGTCTTTCAGCAAGGACTGG Reverse: CCACTTAACTATCTTGGGCTGTG Probe: FAM-TCACATGGTTCACACGGCAGGCAT-BHQ-1	***β2-microglobulin***

ATO: Arsenic trioxide; AUR: Auraptene

## Conclusion

Obtained findings indicated, for the first time, increased cytotoxicity of AUR and ATO when used in combination, which was confirmed by cellular accumulation in the sub G1 phase and down-regulation of *NF-κB* (*REL-A*), *CD44*, *c-MYC*, and *BMI-1*. Hence, combinatorial use of AUR and ATO could be considered to design novel chemotherapy regimens for ATLL.

## Authors’ Contributions

MK, HK, ZD, FK, and SG Doing experiments; SG, AM, and HR Manuscript drafting; MI Research advisors, preparing experimental facilities and kits; FB and HR Research director, conception and design of the study, data analysis; All authors have read and approved the final manuscript.

## Ethical Disclosure

This work does not contain any studies with human participants or animals.

## Availability of Data and Material

The data that support the findings of this study are included in the manuscript and are available from the corresponding author upon reasonable request.

## Conflicts of Interest

This study was financially supported by the Vice-Chancellor for Research and Technology, Mashhad University of Medical Sciences, Mashhad, Iran, under Grant [MUMS 940778]. The authors declare that they have no conflicts of interest.
